# The relationship between gonadotropin releasing hormone and ovulation inducing factor/nerve growth factor receptors in the hypothalamus of the llama

**DOI:** 10.1186/s12958-018-0402-6

**Published:** 2018-08-31

**Authors:** Rodrigo A. Carrasco, Jaswant Singh, Gregg P. Adams

**Affiliations:** Department of Veterinary Biomedical Sciences, Western College of Veterinary Medicine, University of Saskatchewan, 52 campus drive, Saskatoon, Saskatchewan S7N5B4 Canada

**Keywords:** GnRH, TrkA, p75, Llamas, Hypothalamus, Ovulation-induction factor, Nerve growth factor

## Abstract

**Background:**

A molecule identical to nerve growth factor, with ovulation-inducing properties has been discovered in the seminal plasma of South American camelids (ovulation-inducing factor/nerve growth factor; OIF/NGF). We hypothesize that the ovulatory effect of OIF/NGF is initiated at the level of the hypothalamus, presumably by GnRH neurons. The objective of the present study was to determine the structural relationship between GnRH neurons and neurons expressing high- and low-affinity receptors for NGF (i.e., TrkA and p75, respectively) in the hypothalamus.

**Methods:**

Mature llamas (*n* = 4) were euthanized and their hypothalamic tissue was fixed, sectioned, and processed for immunohistochemistry on free-floating sections. Ten equidistant sections per brain were double stained for immunofluorescence detection of TrkA and GnRH, or p75 and GnRH.

**Results:**

Cells immunoreactive to TrkA were detected in most hypothalamic areas, but the majority of cells were detected in the diagonal band of Broca (part of the ventral forebrain) and the supraoptic nuclei and periventricular area. The number of cells immunoreactive to p75 was highest in the diagonal band of Broca and lateral preoptic areas and least in more caudal areas of the hypothalamus (*p* < 0.05) in a pattern similar to that of TrkA. A low proportion of GnRH neurons were immunoreactive to TrkA (2.5% of total GnRH cells), and no co-localization between GnRH and p75 was detected. GnRH neuron fibers were detected only occasionally in proximity to TrkA immunopositive neurons.

**Conclusions:**

Results do not support the hypothesis that the effect of OIF/NGF is driven by a direct interaction with GnRH neurons, but rather provide rationale for the hypothesis that interneurons exist in the hypothalamus that mediate OIF/NGF-induced ovulation.

## Background

As induced ovulators, llamas and alpacas ovulate in response to copulation [1, 2]. The physical stimulation of coitus, however, is not the primary trigger for ovulation, as initially proposed, but rather ovulation occurs in response to a factor present in seminal plasma that induces a preovulatory LH surge [3–6]. The seminal ovulation-inducing factor (OIF) is a potent stimulator of LH release [4, 7], and is capable of inducing ovulation in llamas and alpacas at dose 1/100th of that present in a normal ejaculate [8]. Mass spectrometry and protein crystallography allowed the identification of OIF as ß-nerve growth factor [6] and will be herein-after referred to as OIF/NGF (seminal plasma derived NGF). In a study designed to determine the mechanism by which OIF/NGF elicits LH release from the pituitary gland [9], llamas pretreated with a GnRH receptor antagonist and subsequently treated with OIF/NGF failed to have a preovulatory LH surge. While the main site of action of OIF/NGF in vivo appears to be at the level of the hypothalamus, treatment of primary cell cultures of alpaca, cattle and rat pituitaries with OIF/NGF or seminal plasma also induced LH release [10, 11].

Ovarian follicular development in llamas and alpacas occurs in a wave-like pattern [12], as described in other farm animals [13, 14]. As a monotocous species, each follicular wave involves development of a single dominant follicle which, in llamas and alpacas, is capable of ovulating when it is ≥7 mm in diameter [12, 15]. In the absence of mating, ovulation does not occur, a corpus luteum does not develop, and successive follicular waves emerge at periodic intervals. In contrast, spontaneous ovulators ovulate irrespective of mating, a corpus luteum is present during the majority of the estrous cycle, and progesterone plays a role in follicle maturation and oocyte competence [16]. Ovarian estradiol is not associated with positive feedback on the hypothalamic-pituitary axis to elicit the LH surge in induced ovulators, as it is in spontaneous ovulators, but it does modulate pituitary LH secretion in OIF/NGF-treated llamas [17].

Nerve growth factor is a molecule that has the effect of maintaining and enhancing neuron survival [18], and is present in restricted areas of the central nervous system in rodents, such as the cerebral cortex, the hippocampus, cholinergic pathways in the septal area and the dorsal root ganglia [19, 20]. As well, NGF receptors have been detected by autoradiography in the diagonal band of Broca, caudal putamen, lateral preoptic area and globus palidus in rats [21]. The effect of NGF is mediated through interaction with two different receptors, TrkA and p75. TrkA (also known as NTRK1) is a specific high-affinity receptor that mediates most of the classical actions of NGF, whereas p75 (also known as NGFR) is a less specific low-affinity receptor that has been associated with NGF-activated cell death in oligodendrocytes [22, 23]. Despite the apparent opposing effects mediated by the two receptors, a large proportion of cells in the septal/diagonal band of Broca region [24, 25] and the nucleus basalis [25] bear both p75 and TrkA (mRNA or protein). Pharmacological blockade of TrkA eliminated most of the effects of NGF [26], and p75 is capable of binding to neurotrophins other than NGF (Reviewed by [27]); hence, it is likely that the ovulation-inducing effect of OIF/NGF is driven by interaction with the high-affinity receptor, TrkA.

GnRH secretion is a fundamental signal for the preovulatory LH surge [28]. In an initial effort to address the hypothesis that OIF/NGF induces ovulation through direct interaction with GnRH neurons in llamas, the objective of the present study was to determine if the high- and low-affinity receptors of OIF/NGF are expressed in GnRH neurons of llamas.

## Methods

### Animals and tissue collection

Non-pregnant, non-lactating adult female llamas (*n* = 4) weighting 100 to 140 kg were euthanized using an overdose of pentobarbital during summer (Euthanyl Forte, Bimeda MTC Animal health Inc., Cambridge, Ontario, Canada). The head was separated and immediately perfused via the carotid arteries with 2 l of cold heparinized (10,000 IU Na heparin/L) saline (0.9% NaCl) solution, followed by 2 l of a solution of 4% formaldehyde in phosphate buffered saline (PBS; 0.1 M, pH = 7.4). After the brain was extracted from the cranium, a piece of tissue containing part of the septum and the hypothalamus was dissected out and immersed in the same fixative overnight at 4 °C. The next day, tissues were washed 3 times in PBS and stored in PBS with 0.1% sodium azide at 4 °C until cryoprotection. Samples were immersed in cryoprotectant solution (30% sucrose in PBS) until the tissues sank, and then frozen at − 80 °C until sectioning. Tissues were sectioned transversely (coronal plane) at a thickness of 50 um using a cryostat, and each section was stored in a mixture of 30% sucrose and 30% ethylene glycol in PBS at − 20 °C until immunostaining. Single and double immunohistochemistry was carried out on free-floating sections to optimize staining of thick (50 μm) sections (see below). Animal procedures were approved by the University of Saskatchewan Committee on Animal Care in accordance with guidelines of the Canadian Council on Animal Care.

### Single immunohistochemistry

Anatomical detail and TrkA immunoreactivity were assessed by light microscopy, using adjacent sections stained with Cresyl violet or by immunohistochemistry against TrkA [29]. Briefly, sections were rinsed in PBS and incubated in 3% hydrogen peroxide to block endogenous peroxidases. Sections were heated to 90 °C in sodium citrate buffer for 30 mins (0.1 M; pH = 6.0; Sigma) and incubated in blocking buffer for 1 h. TrkA antibody was applied at 1:500 dilution in blocking buffer for 24 h, sections were rinsed three times in PBS, and goat anti-rabbit antibody conjugated to horseradish peroxidase was used to detect the antigen-antibody complex [29]. Sections were washed three times in PBS, immersed in a solution of DAB for 10 min, rinsed in PBS, mounted on glass slides, air dried and cover-slipped until examination. Anatomical organization was determined using the aid of the *Lama glama* brain atlas of the University of Wisconsin, Madison (http://brainmuseum.org/) and stereotaxic atlases of other mammals [30–32].

### Double immunofluorescence

Two sets of ten equidistant sections per brain (approximately one every 1500 um) were selected for double immunofluorescence labelling for either GnRH with TrkA or GnRH with p75. After removing the cryoprotectant solution, sections were rinsed 4 times in PBS for 10 min each. To expose epitopes to antibodies in tissue sections (Antigen retrieval), samples were heated at 80 °C for 35 min in sodium citrate solution (0.1 M; pH = 6.0; Sigma). After cooling to room temperature, section non-specific binding was blocked with 0.5% BSA 0.5% triton X-100 in PBS for 3 h. Sections were incubated for 48 h at 4 °C in a cocktail consisting of two primary antibodies diluted in 0.5% BSA (Sigma), 0.5% triton x-100, and 0.1% sodium azide in PBS. For both sets of sections (TrkA and p75), anti-GnRH antibody (mouse anti-GnRH SMI 41; Sternberger Monoclonals; Cedarlane, Burlington, Ontario, Canada) was used at a dilution of 1:10,000 in blocking buffer. Anti-TrkA (rabbit anti-TrkA, Santa Cruz biotechnologies; Dallas, Texas, USA) was used at a dilution of 1:500, and rabbit anti-p75 (gift from Dr. Louis F Reichardt, University of California San Francisco, USA) was used at a 1:5000 dilution. Sections were washed 3 times with PBS and incubated in a mixture of secondary antibodies consisting of goat anti-rabbit antibody conjugated to biotin (1:500 for TrkA, 1:1000 for p75; Life Technologies; Burlington, Ontario, Canada) and goat anti-mouse antibody conjugated to Alexa 546 (1:500; Life Technologies; Burlington, Ontario, Canada) for 2 to 3 h at 37 °C in blocking buffer. After washing the secondary antibodies, samples were incubated with streptavidin conjugated to Alexa 488 diluted in blocking buffer (1:200 for TrkA, and 1:5000 for p75; Life Technologies; Burlington, Ontario, Canada) for 1 to 2 h [29]. Finally, sections were washed and mounted on poly-L-lysine coated slides, air dried, incubated for 10 min in a solution of 0.3% sudan black in 70% ethanol (to reduce autofluorescence), air dried again, covered with Vectashield mounting medium (Vectorlabs, Burlington, Ontario, Canada) containing DAPI, and a coverslip was applied. Coverslipped sections were stored at 4 °C in the dark until examination.

Cell numbers were counted manually by a single observer using a wide-field fluorescent microscope at 20× magnification (Zeiss Axioskop 40; Thornwood, New York, USA). To avoid double counting and overestimation, only those cells that displayed a single distinguishable nucleus were counted. Confocal microscopy was performed on a Leica LSM confocal microscope (Leica Microsystems, Concord, Ontario, Canada) with lasers for excitation of Alexa 488, Alexa 546, and DAPI. Stacks were obtained by using a 63× oil immersion objective lens, with a numerical aperture of 1.4. Optical section thickness was 0.7 μm.

### Antibody controls

**Fig. 1:**
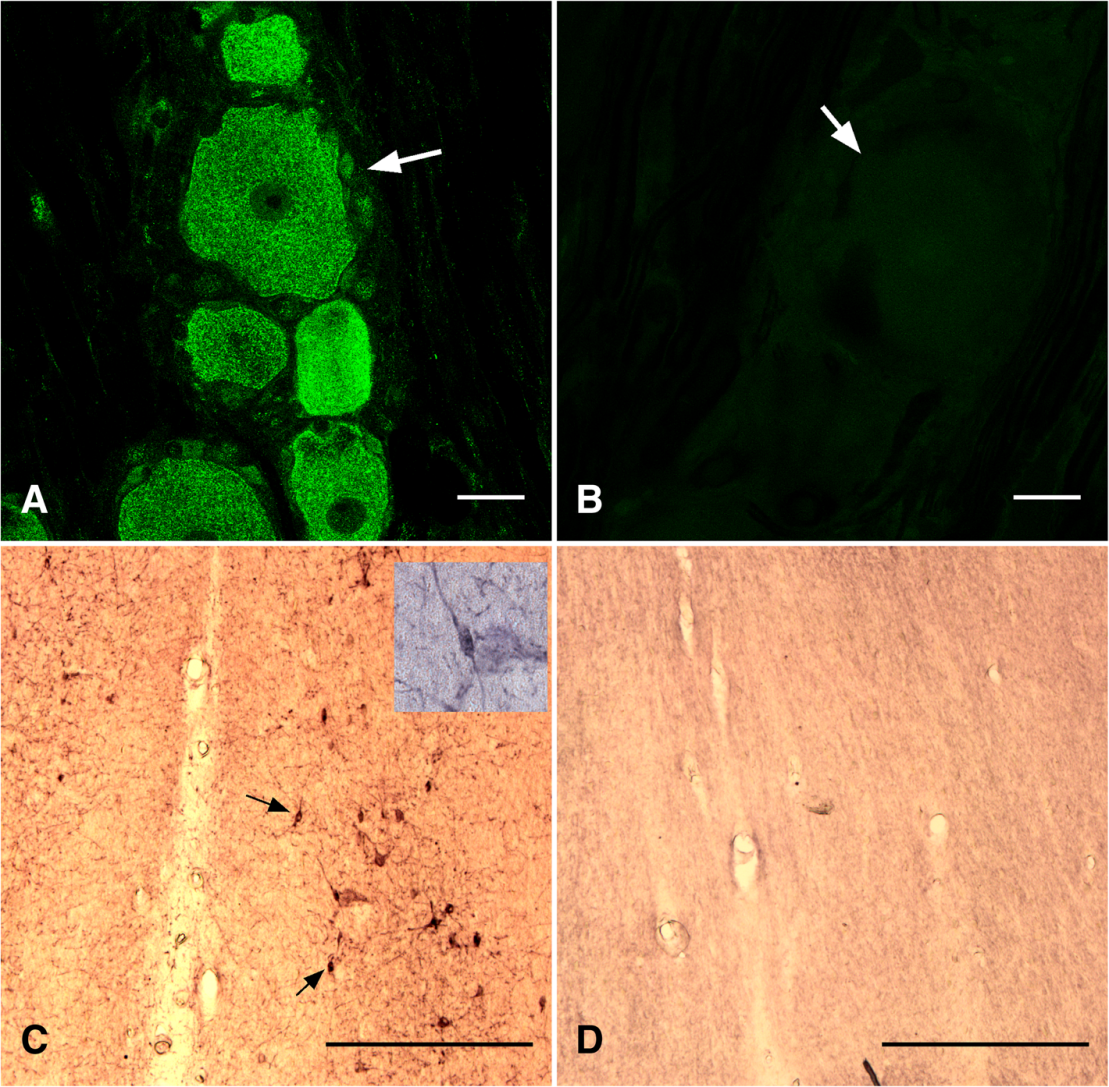
Validation of antibodies against TrkA (**a** positive control; **b** negative control) and p75 (**c** positive control; **d** negative controls) using sections of a dorsal root ganglium (for TrkA) or medial septum (for p75) of a llama. For the negative control sections, primary antibodies were pre-absorbed with the corresponding immunogen. **a**, **b**. Scale bar = 30 um; **c**, **d** 50 um

The TrkA antibody was raised in rabbit against a fragment of the C terminus of human TrkA receptor. Pre-adsorption of the primary anti-TrkA antibody with TrkA immunogen (Santa Cruz Biotechnologies; Dallas, Texas, USA) was performed in a 1 to 5 ratio (protein content) with no resultant immunodetection. Llama dorsal root ganglia were used as a positive control (Fig. 1). GnRH is highly conserved among species [33] and use of the anti-GnRH antibody has been validated previously with different species (rat, [34]; sheep, [35]. We have tested the specificity of the GnRH antibody by pre-adsorption with the GnRH peptide (ab 120184; Abcam, Cambridge, MA, USA) and by replacing the primary antibody with a mouse isotype (IgG 1), both procedures resulted in no immunoreaction. The p75 antibody was raised against the extracellular domain of rat p75 receptor. Anti-p75 antibody specificity was tested by omission of the primary antibody and by preincubating with 5 μg of a fragment containing the extracellular domain of the recombinant human protein (ab157276, Abcam, Cambridge, MA, USA), with no resultant immunoreaction [36].

### Data analysis

Data are expressed as mean ± SEM or as a percentage of the total number of cells displaying double immunoreactivity. The number of GnRH (from both set of double-stained sections), TrkA, and p75 immunopositive cells was compared among anatomical areas by analysis of variance for repeated measures. The total number of cells per brain (GnRH vs TrkA, and GnRH vs p75) were compared by t tests. Differences were considered significant with a *p*-value ≤0.05.

## Results

### Distribution of TrkA immunoreactive cells

Llama dorsal root ganglia stained against TrkA receptor showed a strong immunoreaction (Fig. 1a). The signal was restricted to sensory neurons; no reaction was detected in satellite cells of the dorsal root ganglia. When the antibody was pre-incubated with TrkA peptide, no signal was detected (Fig. 1b), documenting the specificity of the antibody signal. The immunoreactivity was restricted primarily to the cytoplasm surrounding the neuronal nuclei, whereas no identifiable neuronal projections (fibers) were detected.

**Fig. 2:**
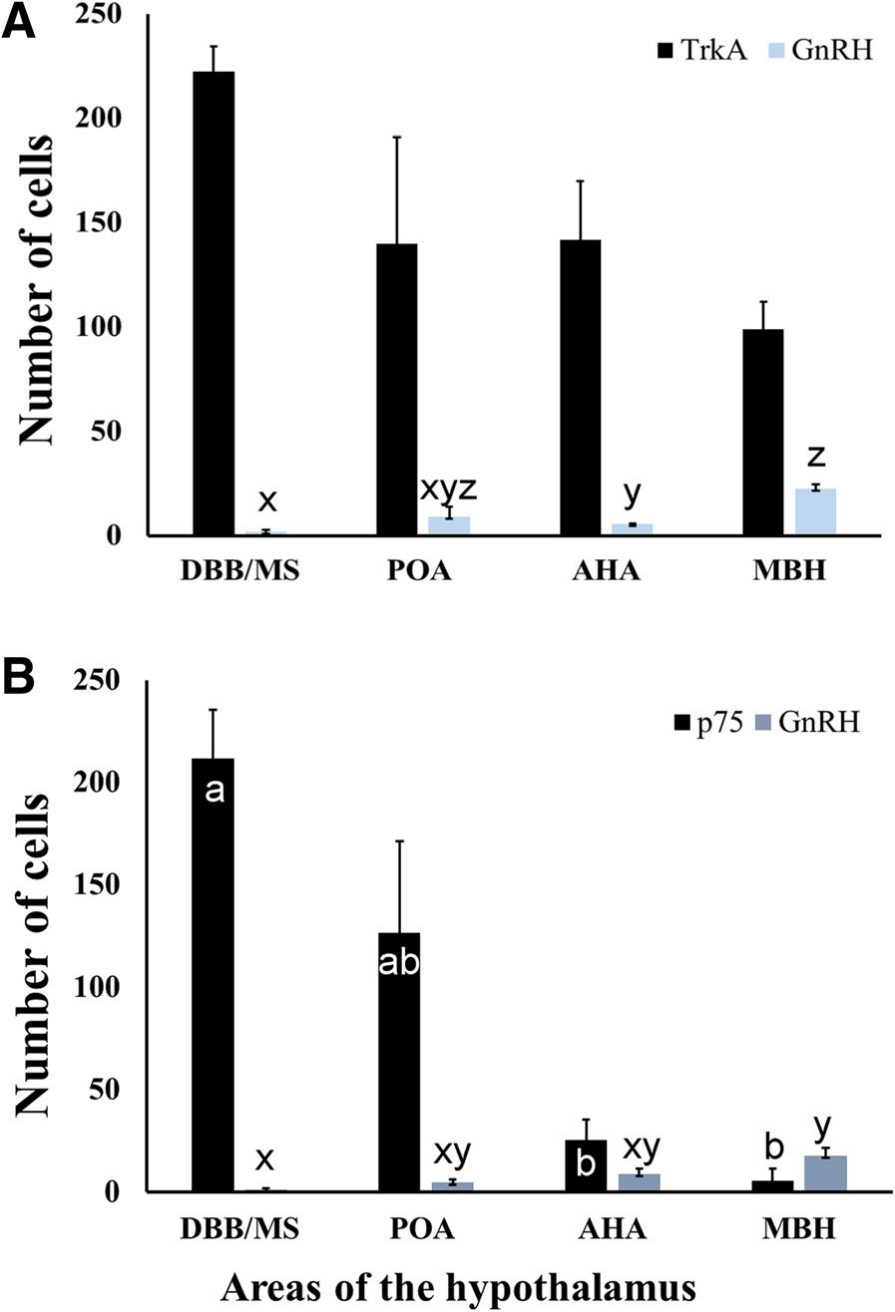
Distribution of cells expressing immunoreactivity to GnRH, TrkA, and p75 (mean ± SEM number of cells) in the hypothalamus and preoptic areas of llama brains (*n* = 4). Double staining was done on separate sets of sections; hence, cell numbers for TrkA and GnRH are presented in (**a**), and cell numbers for P75 and GnRH are presented in (**b**). DBB/MS: diagonal band of Broca/medial septum, POA: preoptic area, AHA: anterior hypothalamic area, MBH: medio-basal hypothalamus. ^ab^ For TrkA and p75 neurons, values with no common superscript among regions are different (*p* < 0.05). ^xyz^ For GnRH neurons, values with no common superscript among regions are different (*p* < 0.05)

**Fig. 3:**
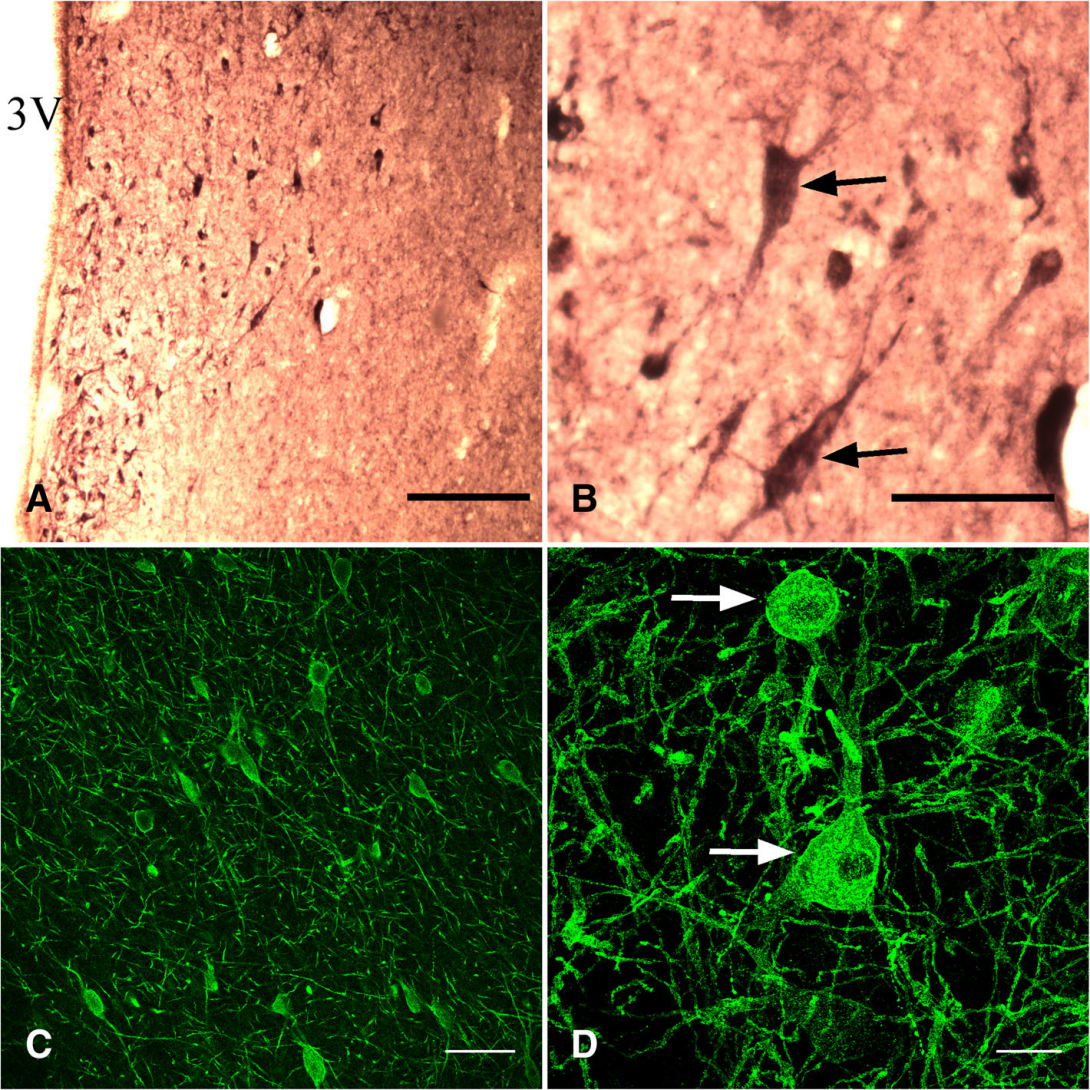
Immunoreactivity to TrkA and p75 receptor in the hypothalamus of llamas. Low (**a**) and high (**b**) magnification of TrkA immunoreactive neurons (arrows) in the periventricular area, as detected by light microscopy. Note that B is a magnification of A. P75 immunoreactive neurons (arrows) in the septal area at low (**c**) and high magnification (**d**), as detected by immunofluorescence. 3 V: third ventricle. Scale bars: **a** 200 μm., **b** 50 μm., **c** 100 μm., **d** 30 μm

TrkA immunoreactivity was present in all hypothalamic areas and nuclei examined, except in the median eminence, dorsal hypothalamus, and the optic chiasma. The variation in the number of TrkA positive cells was sufficiently high that no significant difference was detected among the immunoreactive areas, but was numerically greatest in the area of the diagonal band of Broca and medial septum, and least in the medio-basal hypothalamus (Fig. 2a). Importantly, relative accumulations of TrkA immunopositive cells were found in the diagonal band of Broca (part of the ventral forebrain), and the supraoptic and periventricular areas (part of the anterior hypothalamus and mediobasal hypothalamus; Fig. 3). TrkA immunoreactive neurons were only occasionally detected in the arcuate nucleus and retrochiasmatic area (data not shown).

### Distribution of p75 immunoreactive neurons

A dense network of p75 immunoreactive fibers and cells was detected in the ventral forebrain (i.e., diagonal band of Broca and medial septum), decreasing caudally (*p* < 0.05) in a pattern similar to that of TrkA (Fig. 2b). Low immunoreactivity was found in the medio-basal hypothalamus and none in the mammillary hypothalamus. An abundance of immunoreactive cells was found in the bed nucleus of the stria terminalis, surrounding the anterior commissure in the preoptic area. Strong immunoreactivity was detected in the ependymal cells of the lateral and third ventricles. Individual fibers were detected in proximity to the lateral ventricle and a dense network of fibers appeared in the organum vasculosum and the ventral aspect of the third ventricle at the level of the arcuate nucleus and median eminence. Representative images of P75 neurons are shown in Fig. 3c-d.

### Morphological relationship between NGF receptors and GnRH

**Fig. 4:**
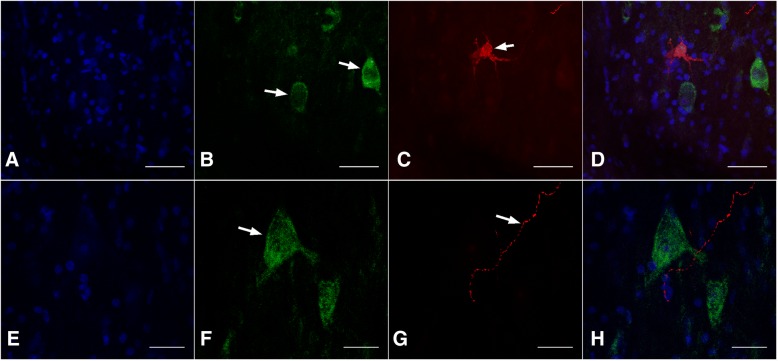
Immunoreactivity to GnRH and TrkA in the llama hypothalamus, detected by double immunofluorescence. The upper panel (**a**-**d**; maximum intensity projections) shows the relationship between TrkA neurons (**b**; arrows; green) and GnRH neurons (**c**; arrow; red). The lower panel (maximum intensity projections) illustrates the relationship between TrkA neurons (**f**; arrow) and GnRH fibers (**g**; arrow). Images **d** and **h** depict the overlay of the corresponding panels (**a**-**d** and **e**-**h**) including the nuclear counter-stain (**a**, **e**). Scale bars: **a**-**d** 30, E-H 50 μm

Overall, TrkA and p75 immunoreactive cells were found in abundance in the llama hypothalamus (2390.2 ± 131 and 1097.5 ± 209.7 cells, respectively), and GnRH cell counts did not differ between slides co-stained with TrkA vs p75 (40.5 ± 7.3 vs 33.7 ± 10.5 cells, respectively; *p* = 0.68). Of the total number of cells (among 4 animals) in the hypothalamus displaying immunoreactivity to GnRH, 156/160 (97.5%) stained for GnRH alone, and 4/160 (2.5%) stained for both GnRH and TrkA. Of the number of cells displaying immunoreactivity to TrkA in the hypothalamus of 4 animals, 9477/9481 (> 99%) stained for TrkA alone; i.e., only 4/9481 (0.042%) stained for both TrkA and GnRH. Aside from the low degree of co-localization, TrkA and GnRH neurons were not commonly visualized in the same anatomical plane, and on only three occasions appeared closely related (i.e., within the same microscopic field; Fig. 4a-d). In the three instances where GnRH immunoreactive fibers were found in close relationship to TrkA immunoreactive cells, there was no apparent contact (Fig. 4e-h).

**Fig. 5:**
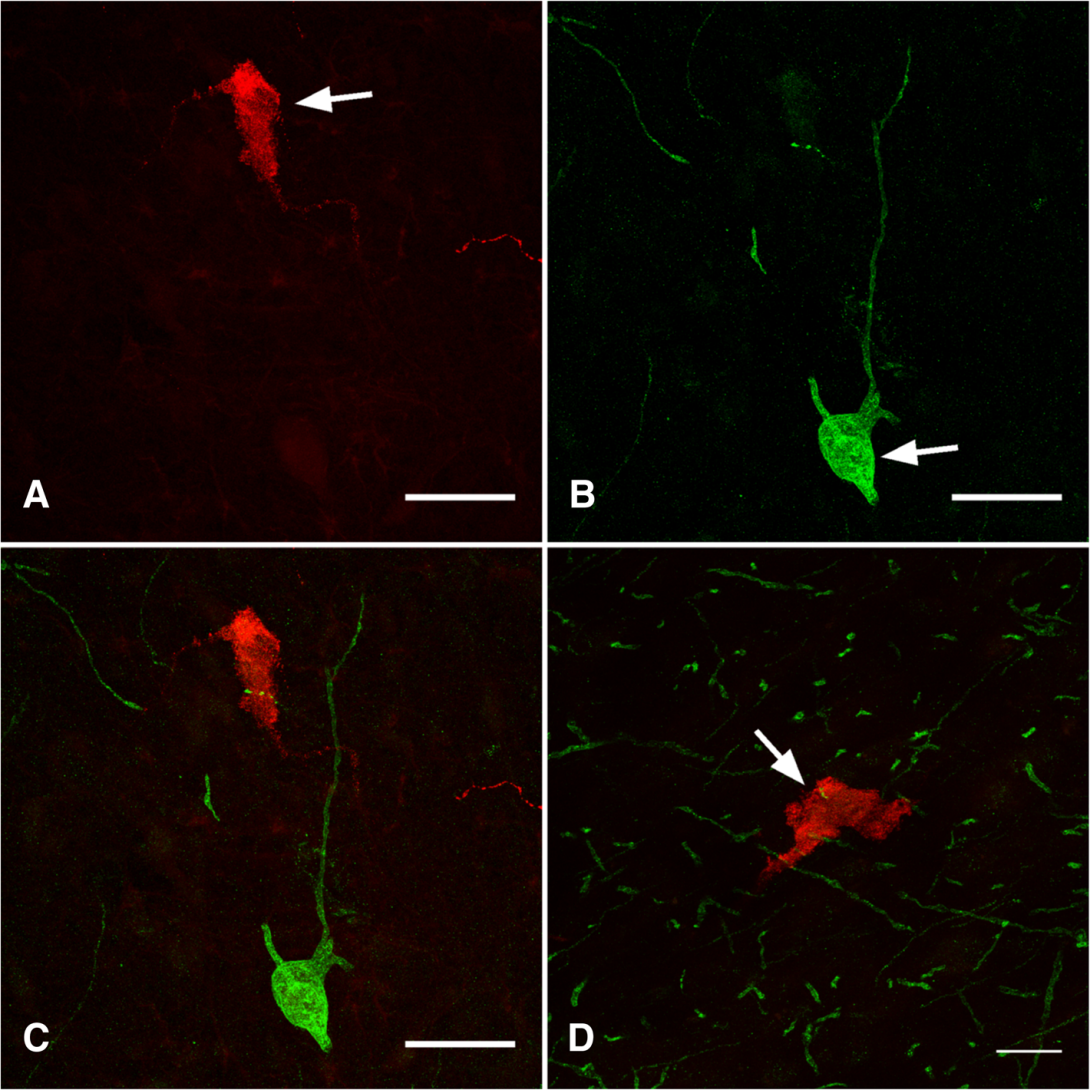
Immunoreactivity to GnRH and p75 in the llama hypothalamus, detected by double immunofluorescence. A single microscopic field (**a**-**c**; maximum intensity projection displaying cells immunoreactive for GnRH (red), p75 (green), and both channels (merged). A GnRH immunoreactive neuron (**d**, red) in proximity to p75 immunoreactive fibers (D, green). Arrows indicate nerve cell bodies. Scale bars: **a**-**c**, 50 μm. **d** 30 μm

**Fig. 6:**
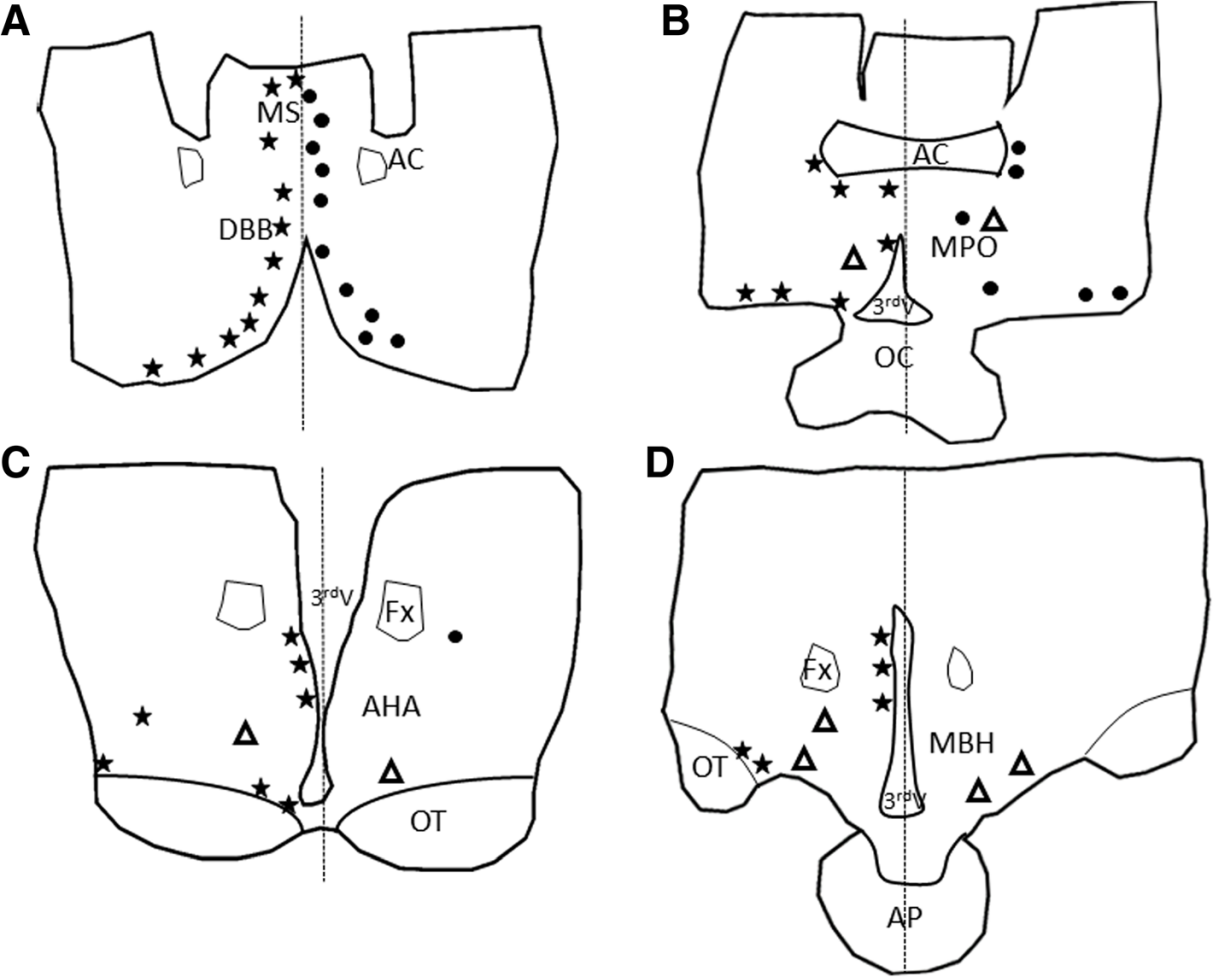
A representation of the distribution of TrkA (stars), p75 (dots), and GnRH (triangles) neurons in the hypothalamus of llamas. Tracings were made from transverse (coronal) sections, progressing from cranial to caudal, of the diagonal band of Broca/medial septum (**a**), preoptic area (**b**), anterior hypothalamus (**c**), and medio-basal hypothalamus (**d**). For a given histological section, each symbol represents approximately 10 neurons for TrkA and p75, and 5 neurons for GnRH. Each diagram is divided at the midline (dashed line) to permit representation of TrkA on the left and p75 on the right. MS: medial septum, DBB: diagonal band of Broca, MPOA: medial preoptic area, AC: anterior commissure, OC: optic chiasma, Fx: fornix, AHA: anterior hypothalamic area, OT: optic tract, MBH: medio-basal hypothalamus, 3^rd^V: third ventricle, AP: anterior pituitary

GnRH and p75 immunoreactivities were never detected in the same cell. The two cell types were occasionally located in the same microscopic field (Fig. 5a-c), but the majority of the respective neurons were detected in different areas. P75 neurons were present in greater numbers in the septal area and anterior areas of the hypothalamus (i.e., preoptic area), and virtually non-existent in posterior areas (i.e., mammillary hypothalamus). GnRH neurons were located mostly in the medio-basal hypothalamus and were generally sparse. GnRH cell counts did not differ between slides co-stained with TrkA vs p75 (*p* = 0.68). P75 immunoreactive fibers were occasionally located in proximity to GnRH neurons (Fig. 5d). A scheme of the relationship between GnRH, TrkA and p75 among examined sections is shown in Fig. 6.

## Discussion

Ovulation-inducing factor has been shown to induce ovulation in a high proportion of llamas and alpacas after parenteral administration. The effect appears to be mediated via GnRH neurons (directly or indirectly) resulting in preovulatory LH secretion from the gonadotropes in the anterior pituitary [9]. Results of the present study do not support the hypothesis that OIF/NGF effects a response through direct interaction with GnRH neurons in llamas since the high- and low-affinity receptors for OIF/NGF were detected in 2% and 0% of GnRH neurons, respectively. It is unlikely that such a low proportion of GnRH neurons would drive the preovulatory LH surge. In mice and sheep, for instance, detection of the c-FOS proto-oncogene (a marker of neuronal activation) revealed that about 40% of GnRH neurons are activated during the LH surge [37–39]. Similar to the triggering factor for the LH surge in spontaneous ovulators (estradiol), the triggering factor in camelids (OIF/NGF) must involve an intermediate cell type to interact with GnRH neurons (e.g., kisspeptin neurons, norepinephrine neurons). This view is consistent with the notion that GnRH neurons act as a final output for a complex interplay between neurons [40].

In the present study, TrkA immunoreactive cells were present in most areas examined but were accumulated in three major structures: 1) the diagonal band of Broca in the ventral forebrain, 2) the supraoptic nucleus, and 3) the periventricular area of the third ventricle. These findings are similar to those of studies in rats and macaques [25, 41]. The presence of TrkA immunoreactive cells in the periventricular area offers interesting insight into the route of action of the OIF/NGF system. This area is in close contact with the third ventricle and if OIF/NGF crosses the blood-brain barrier into the cerebrospinal fluid, it would be available to interact with the TrkA receptors in regions of the brain that influence GnRH neurons. In an autoradiographic study of rats, radiolabelled NGF injected into the cerebral ventricle was detected in a layer of the parenchyma surrounding the lateral, third and fourth ventricles [42], consistent with the idea that the ependymal epithelium lining the third ventricle is permeable to such molecules.

Whether NGF crosses the blood-brain barrier in llamas remains unknown. In mice, both the high molecular weight form (7 s NGF) and the low molecular weight form (2.5 s NGF or β-NGF) were detected in the central nervous system after radiolabelling and intravenous administration [43]. With a molecular weight of 26 kDa [6], it seems unlikely that OIF/NGF can simply diffuse through the blood brain barrier [44]. Thus, we hypothesize two routes of NGF entry into the brain: 1) by interacting with the choroid plexus with subsequent active secretion into the cerebrospinal fluid, or 2) by interacting with neurons or their projections in the vicinity of the organum vasculosum and the median eminence (reviewed by [45, 46]). If OIF/NGF crosses the blood-brain barrier by interacting with the epithelium of the choroid plexus, the expression of a specific receptor is required. Such a mechanism has been theorized as one of the pathways for leptin, a 16 kDa hormone produced by adipocytes, which exerts a suppressive effect on appetite possibly via the choroid plexus to act in the arcuate nucleus [47, 48]. Although the low-affinity receptor, p75, has been identified in the rat choroid plexus [49], as well as mRNA of NGF and other neurotrophins, the high affinity receptor, TrkA, has not been detected at high levels in the choroid plexus [50]. Hence, the expression of NGF receptors and their association with a transport system in the choroid plexus of llamas remain unknown.

Circumventricular organs, including the organum vasculosum and the median eminence in the hypothalamus, are areas of the brain where the blood-brain barrier is modified, allowing the secretion of peptides/proteins or sensing the internal milieu by neurons [45]. Thus, the presence of receptors in these structures would allow interaction with neurotrophins circulating in the vascular system. Consistent with studies of other species [51], we found abundant expression of the low-affinity receptor in the diagonal band of Broca/medial septum, lateral preoptic area and circumventricular organs (organum vasculosum and median eminence). The expression of the low affinity receptor in the median eminence and ependymal layer could be the reflex of important physiological functions. It has been shown that the administration of fluorescent leptin labels the ependymal/tanycyte layer as soon as 15 min after treatment, and the mechanism is associated to the leptin receptor [52]. Given the ability of p75 to bind to several neurotrophins besides NGF (reviewed in [53]), the presence of p75 in circumventricular organs may be attributed a role in mediating neurotrophin entry into the brain or activating signaling pathways. Based on results of PC_12_ neuronal culture experiments, the p75 receptor is capable of internalizing NGF independent of TrkA [54]. However, it remains to be determined if p75 receptor-expressing cells are involved in OIF/NGF-induced ovulation in South American camelids.

Kisspeptin has been documented as an important mediator of GnRH neuron function in several species, such as laboratory rodents [55], sheep [56], and monkeys [57]. In the musk shrew, an induced ovulator, mating activated kisspeptin neurons in the preoptic area, and administration of kisspeptin mimicked mating-induced ovulation [58]. Although no data were reported, authors of a recent review on camelids speculated that kisspeptin may be a mediator of OIF/NGF-induced ovulation [59]. Concentrations of kisspeptin neurons have been identified in the preoptic area and in the arcuate nucleus, and these two populations display different functions in mediating tonic or surge secretion of GnRH [60]. The role of kisspeptin neurons in the ovulatory mechanism in camelids remains to be elucidated.

## Conclusion

Results of the present study do not support the hypothesis that OIF/NGF interacts directly with GnRH neurons through receptors to elicit ovulation in llamas. The presence of both high- and low-affinity receptors in the hypothalamus of llamas provides rationale for the hypothesis that OIF/NGF interacts with an interneuron or a group of interneurons that provide inputs to GnRH neurons. The neurochemical identity of the TrkA and p75 immunoreactive cells in the hypothalamus of llamas, however, remains to be established.

## Abbreviations

BSA: Bovine serum albumin
GnRH: Gonadotrophin-releasing hormone
LH: Luteinizing hormone
OIF: Ovulation-inducing factor
OIF/NGF: Ovulation-inducing factor/nerve growth factor
PBS: Phosphate buffer saline
